# Introduction for the follow-up of the Eighth International Kraepelin Symposium at LMU Munich

**DOI:** 10.1007/s00406-023-01615-9

**Published:** 2023-05-18

**Authors:** Schaub Annette, Peter Falkai

**Affiliations:** Department of Psychiatry and Psychotherapy, University Hospital, Ludwig Maximilians University Munich, Nussbaumstr. 7, 803336 Munich, Germany

**Keywords:** Schizophrenia, Borderline personality disorder, Psychoeducational and family therapy, Cognitive behavior therapy, Metacognitive training, Metabolic disturbances, Early intervention, Relapse prevention

This editorial reports from seven international working groups from the US and Europe as follow-up of the Eight Kraepelin Symposium in Munich, LMU, recruiting former and recently recruited ones. It covers basic research investigating the consequences of genetic and environmental factors on psychosis in the translation in preventing psychosis in children of parents with severe mental illness. Secondary prevention aims to avoid further episodes having foci on translation of new mechanisms targeting family communication, patterns of cannabis use or targeting cognitive biases in metacognitive training. Translation in later phases of psychoses refer to translating the knowledge, its evidence and meta-analysis. Novel foci include psychoeducational groups for close relatives of patients with borderline personality disorder and lifestyle behaviors combined with metabolic disturbances in a transdiagnostic psychiatric sample. Recently, metacognitive training gained importance being an important addition to family therapy and cognitive behavioral therapy. Strategies that proved their effectiveness are to be implemented in standard treatment as well as strategies implemented to train professionals in effective crisis and family management.

In the nineteenth century, Emil Krapelin [1] pioneered psychopathology considering the relationship between physical and mental states. He also funded this hospital, nowadays, called the Department of Psychiatry and Psychotherapy, University Hospital, LMU Munich. It was the site for our Kraepelin symposiums led by Peter Falkai and Annette Schaub [2, 3] influenced by the then existing pandemic that currently subsided.

This special supplement related to the Eighth Kraepelin Symposium—Translation in Psychiatry and Psychotherapy—A Life-long Necessity [3] covers the input of 7 working groups 3 overlapping with 2020. They come from the United States and Europe and focus on areas of interest in life-span covering primary, secondary and tertiary prevention. There are four chapters summarizing the available knowledge, gaps in respective fields, and ideas for future research.

Translation from mechanism to mental health practices covers basic research in psychological targets in trauma and its translation in mental health practices [4]. Translation of new mechanisms in secondary prevention focus on family communication [5], cannabis patterns in first episode [6] or cognitive biases in metacognitive training [7] as well as on evidence advancing understanding and treatment in later phases of psychoses based on meta-analysis [8]. Pioneering psychoeducational groups in borderline personality disorder [9] and metabolic disturbances and weight gain in a transdiagnostic sample [10] are novel topics.

The vulnerability–stress-coping model (2–3.5) is our conceptual framework guiding psychoeducational and family therapy focusing on the interaction between biological vulnerability, protective personal and environmental factors Garosi et al. and the real world FACE-SZ cohort [4] investigate the impact of psychological targets in trauma in parent ‘s history of severe mental illness (PHSMI) on schizophrenia outcome. 10.7% of 724 patients were classified in this group. Its translation in mental health practices includes the development of specific public health prevention programs protecting children from pejorative psychiatric outcomes. Hahlweg and Baucom [7] stress the importance of family therapy and its combination with psychopharmacotherapy. Research on the expressed emotion concept (EE) showed an increase of relapse rates by a factor of 2.5 for patients returning to a high-EE compared to a low-EE-family and a significant reduction in relapse from 49 to 13% comparing standard treatment to family therapy (FT). More than 50 RCT showed its effectiveness in various culturally diverse countries including FTl and antipsychotics. However, despite these strong data, family involvement is still under-implemented in mental health care asking for more research and training for professionals to fill this gap.

Wright et al. [8] investigate the relationship between patterns of cannabis use in first-episode psychosis (FEP) and its functional and symptomatic trajectories. There is heterogeneous data on cannabis use predicting outcome, however, sporadic user who received early intervention therapy (EIS) improved more in overall psychosocial functioning than those in clinical care. Consistent users showed no difference in their trajectories to never users. Individuals using cannabis sporadically showed less clinical improvement than non-users, however, participants in early interventions reduced the negative effects of sporadic cannabis use on clinical outcomes. Meta-cognitive training for psychosis (MCT) started 2 decades ago [7]. Focusing on primary targets such as metacognitive variables and decision confidence led to major changes. New modules addressing mood and sustainability of effects via homework exercises, a smartphone and culturally sensitive language versions enhanced dissemination. Several meta-analyses on the efficacy of MCT (positive symptoms, insight, cognitive biases) led to its inclusion in national treatment guidelines.

Bighelli et al. [8] investigate the effects of psychological treatment on psychosocial functioning in people with schizophrenia providing a systematic review and meta-analysis of 58 randomized controlled trials (5048 participants). CBT and third wave CBT were superior to controls in improving psychosocial functioning, whereas creative or integrated therapies showed no benefit.

Novel foci include psychoeducational groups for close relatives of patients with borderline personality (BPD). Pitschel-Walz et al. [9] evaluated 33 persons in a 10-session program assessing perceived burden, knowledge about the disorder, quality of life and group process. There was a significant decrease of burden, an increase of knowledge about the disorder and a positive feedback if the participants, however, quality of life did not change. Due to the magnitude of overweight ranges, Simon et al. [10] questioned the role of medication and lifestyle factors in a transdiagnostic psychiatric sample assessing biological factors and their predictive capability for weight gain during treatment. In a naturalistic study 4-week follow-up study included 163 transdiagnostic in patients treated with weight gain-associated medication. Drug attitude change interacted with BMI, drug dosage, and presence of metSy predicted weight change. Lifestyle factors, especially eating behaviors, relate to metabolic disturbances and predicted weight gain in interaction with clinical parameters.

In summary, the vulnerability-stress-coping model provides valuable targets for treatment stressing psychoeducation, family therapy and cognitive behavioral therapy (CBT) and recently MCT proved their effectiveness in meta-analysis. Psychoeducation, communication, and problem-solving skills are the main tools for relapse prevention. Novel trends refer to pioneering psychoeducational groups in borderline personality disorder, cannabis patterns in first episodes and predictive capability for weight gain during treatment in a transdiagnostic sample.

Primary intervention is to focus on psychological targets in trauma in children of parents with PHSMI and there should be visiting and complex community programs, residential treatments and online help. Further research, as well as more training for professionals on effective crisis and family management seems necessary.
